# Cross-cultural adaptation and validation of the health-promoting lifestyle profile II for Mongolian university students

**DOI:** 10.3389/fpubh.2026.1846655

**Published:** 2026-06-05

**Authors:** Erdenezul Uitumen, Klára Tarkó

**Affiliations:** 1 Doctoral School of Education, Faculty of Humanities and Social Sciences, University of Szeged, Szeged, Hungary; 2 Department of Nutrition, School of Public Health, Mongolian National University of Medical Sciences, Ulaanbaatar, Mongolia; 3 Juhász Gyula Faculty of Education, Institute of Applied Health Sciences and Environmental Education, University of Szeged, Szeged, Hungary

**Keywords:** health-promoting lifestyle, Mongolia, reliability, university students, validation

## Abstract

**Background:**

University lifestyle can have lasting effects on students’ health, yet culturally validated instruments to examine health-promoting lifestyles are lacking in Mongolia. This study aimed to adapt the Health-Promoting Lifestyle Profile II (HPLP-II) and to assess the validity and reliability of its Mongolian version among students in Ulaanbaatar.

**Methods:**

A cross-sectional study was conducted with 827 undergraduate students from three universities in Ulaanbaatar, Mongolia. The instrument was translated from English into Mongolian using standard forward- and backward-translation procedures. The final version retained six dimensions and 51 items. Construct validity was assessed using confirmatory factor analysis for a six-component structure, and internal consistency was evaluated using Cronbach’s *α* and McDonald’s *ω*.

**Findings:**

Confirmatory factor analysis indicated a good fit for the six-factor structure (*χ*^2^ = 14233.03; df = 1,275; *p* < 0.001; CFI = 0.95, GFI = 0.95, TLI = 0.95, SRM*R* = 0.065, and RMSEA = 0.031). The overall HPLP-II demonstrated high internal consistency (*ω* = 0.92, *α* = 0.91). However, the nutrition dimension exhibited the lowest reliability and factor loadings, suggesting revision for future studies.

**Conclusion:**

The Mongolian version of the HPLP-II demonstrated acceptable reliability and construct validity, supporting the use of a six-dimensional, 51-item instrument to assess health-promoting lifestyles among Mongolian university students. These findings provide a foundation for future health-promotion interventions in Mongolian university settings.

## 1 Introduction

The World Health Organization (WHO) (1) stated that people of all ages are at risk for noncommunicable diseases (NCDs) due to unhealthy lifestyle behaviors, such as physical inactivity, poor diet, alcohol use, and smoking. Health promotion plays an essential role in improving individual well-being and preventing risks associated with NCDs (1). Adopting a healthy lifestyle can prevent disease, reduce premature mortality, and increase life expectancy (2–5). Therefore, assessing health-promoting lifestyles is essential for generating data to guide intervention and support self-management of health behaviors within populations (6).

To measure health-promoting lifestyles effectively, reliable and culturally valid instruments are needed (7, 8). Targeting younger populations and increasing their awareness can improve future health outcomes (9). The Health-Promoting Lifestyle Profile II (HPLP-II), developed and validated by Walker and Hill-Polerecky (6) based on Pender’s Health Promotion Model (10), is one of the most widely used instruments for assessing health-promoting behaviors. The tool consists of 52 items, which examine the frequency of health-promoting lifestyle behaviors within six domains, including health responsibility, nutrition, physical activity, spiritual growth, interpersonal relationships, and stress management (6).

Although the HPLP-II has been cross-culturally validated in numerous non-English speaking countries, including Spain, Portugal, Italy, China, Japan, Malaysia, Jordan, Armenia, Sri-Lanka, Colombia, and Turkey (11–21). Depending on the cultural context, different versions of the HPLP-II have been psychometrically validated with varying numbers of items and varying levels of internal consistency, as measured by Cronbach’s *α*. The original 52-item structure of the HPLP-II was retained in studies conducted in Japan (20), Colombia (21), Portugal (22), and Armenia (23), which all reported acceptable Cronbach’s *α* coefficients ranging from 0.93 to 0.94. The Taiwanese translation of the HPLP-II was conducted by Meihan and Chung-Ngok (15), omitted one item to reach an acceptable Cronbach’s *α* of 0.73. Kuan et al. (14) eliminated two items from the Malay version of the HPLP-II, with Cronbach’s *α* coefficients ranging from 0.74 to 0.88 across the subscales. In Arabic (13) and Turkish (17) translations of the HPLP-II, 48 items were retained, and the scales demonstrated excellent internal consistency (*α* = 0.89–0.92). The Chinese version of the HPLP-II retained 40 items from the original scale, which had an acceptable Cronbach’s *α* of 0.91 (24). The Italian version of HPLP-II was validated as a 26-item with a Cronbach’s *α* of 0.90 (18). To our knowledge, it has not yet been adapted or validated for the Mongolian context. This represents a significant research gap, as culturally adapted instruments are crucial for accurately examining health behaviors and designing effective health-promoting interventions in university settings.

Several studies in Mongolia have utilized multi-dimensional questionnaires to assess healthy lifestyles or health-related behaviors focused on university students. For example, the World Health Organization Quality of Life-Brief Version (WHOQOL-BREF) (25), which measures dimensions such as environmental health, general well-being, physical health, psychological health, and social relationships, was translated into Mongolian and validated by Bat-Erdene et al. (26) in a sample of 301 aged above 18. Only 14.3% of the participants were university students. Given that the university lifestyle can have lasting effects on students’ health (9) there is a need for a validated and reliable instrument specifically for this population. Adapting an instrument for use in different countries and populations requires not only accurate translation but also linguistic and cultural adaptation (27). Thus, the present study aimed to translate and culturally adapt the HPLP-II into Mongolian and to determine its validity and reliability among university students in Ulaanbaatar city. We hypothesize that the use of the validated scale will be relevant for the health intervention and promotion to the targeted population.

## 2 Materials and methods

### 2.1 Study design

A cross-sectional study design was employed, utilizing the self-administered Mongolian HPLP-II questionnaire. Data were collected from April to May of 2024 at three major universities: the Mongolian State University of Education (MSUE), the National University of Mongolia (NUM), and the Mongolian University of Science and Technology (MUST).

### 2.2 Participants

Undergraduate students aged 18 to 24 were recruited through a convenience sampling approach.

University departments and lecturers assisted in recruiting students and in distributing the paper-form survey after their classes. A total of 900 questionnaires were administered, and 827 were completed correctly, yielding a 92.0% response rate. Table 1 presents the sociodemographic characteristics of the university students. Ethical approval was obtained from the IRB of the Doctoral School of Education, University of Szeged (reference number 9/2024).

**Table 1:** The sociodemographic characteristics of the participants (*N* = 827).

Variables		Mean (SD)	Frequency *n* (%)
Age		19.4 (1.37)	
Gender			
	Male		330 (39.9)
	Female		497 (60.1)
University			
	MSUE		251 (30.4)
	NUM		277 (33.5)
	MUST		299 (36.2)
Year of study			
	Year 1		308 (37.3)
	Year 2		188 (22.7)
	Year 3		248 (30.0)
	Year 4 and > 4		83 (10.0)
Location of secondary school			
	Ulaanbaatar		352 (42.6)
	Rural		475 (57.4)
Type of residence			
	Parental home		383 (46.3)
	Dormitory		168 (20.3)
	Rental		153 (18.5)
	Other		123 (14.9)
Working while studying			
	Yes		382 (46.3)
	No		445 (53.8)
Economic status			
	Good		109 (13.2)
	Average		629 (76.1)
	Less than average		89 (10.8)
Body mass index			
	<18.5		88 (10.6)
	18.6–24.9		673 (81.4)
	>25.0		66 (8.0)
Cigarette use			
	Yes		140 (16.9)
	No		687 (83.1)

### 2.3 Measures

The HPLP-II is a multidimensional instrument developed by Walker and Hill-Polerecky (6) to assess the frequency of health-promoting behaviors. The tool consists of 52 items rated on a 4-point frequency scale for responses (1 – never, 2 – sometimes, 3 – often, 4 – routinely). The HPLP-II covers six health dimensions: health responsibility (HR), nutrition (N), physical activity (PA), spiritual growth (SG), interpersonal relationships (IR), and stress management (SM). Construct validity confirmed the six-dimensional structure of the health-promoting lifestyle. Criterion validity of the scale was established by significant correlations (*r* = 0.27–0.50). The internal consistency of the original HPLP-II was acceptable, with a Cronbach’s *α* coefficient of 0.94 for the overall scale of the HPLP-II, and 0.79–0.87 for six dimensions.

### 2.4 Translation procedure

**Figure 1:**
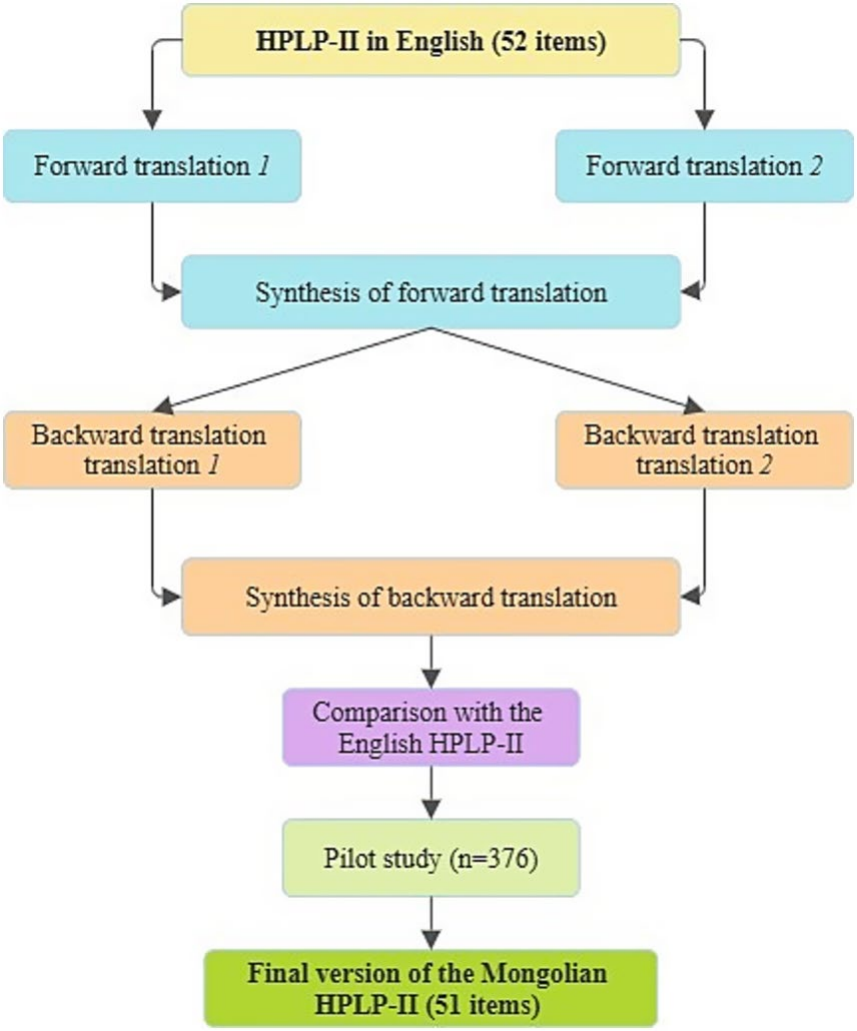
Translation procedure of the HPLP-II into Mongolian.

Figure 1 presents the translation procedure. The HPLP-II was translated into Mongolian, using the standard forward-backward translation procedure, following Beaton et al. guidelines (27), by bilingual health professionals. For cultural adaptation, two semantically similar items in the HR dimension were merged: “*Discuss my health concerns only with health professionals”* and *“Ask health professionals for information on self-care.”* Items of the N dimension were modified to align with the Mongolian national dietary guidelines (28) (Table 2).

**Table 2:** Modification of the nutrition subscale items using the Ger recommendation.

Original HPLP-II item (English)	Translated Mongolian item (Backward-translation)	Rationale for modification
Eat 6–11 servings of bread, …	Eat 9–10 servings of wheat products …	The original range (6–11 servings) was replaced with the precise recommendation (9–10 servings) from the Mongolian Ger recommendation.
Eat 2–3 servings of milk, yogurt, …	Eat 3–4 servings of dairy products …	Dairy foods are an essential product of the traditional Mongolian diet. The Ger recommendation recommends a higher intake (3–4 servings).
Eat only 2–3 servings of meat ….	Eat only 3–4 servings of protein-containing products …	The modification acknowledges the contextualization of protein consumption patterns, adjusting the upper limit (3–4 servings).

The questionnaire was pilot-tested with 376 students. The final version comprised 51 items across six dimensions, each scored on a 4-point frequency scale. The full instrument, including the English backward translation, is provided in the Supplementary material.

### 2.5 Data analysis

Data analyses were conducted using JASP and SPSS 25. Descriptive statistics included frequencies and percentages for sociodemographic characteristics and the distribution of health-promoting patterns among participants and response patterns.

The construct validity of the HPLP-II and its dimensions was confirmed using confirmatory factor analysis (CFA). Factor loadings above 0.35 were considered acceptable indicators (29). The Unweighted Least Squares (ULS) estimation method was employed in the CFA. ULS does not assume multivariate normality and is appropriate for the 4-frequency scale, as it yields accurate item parameter estimates (30, 31). Model diagnostics, including modification indices and standardized residuals, were examined. No post-hoc modifications were conducted to preserve the original six-factor structure.

Acceptable fit index values were: Comparative Fit Index (CFI), Goodness of Fit Index (GFI), and Tucker-Lewis Index (TLI), above 0.90; Root Mean Square Error of Approximation (RMSEA) and Standardized Root Mean Square Residual (SRMR) less than 0.08 (32).

Internal consistency for each dimension of the HPLP-II was assessed with Cronbach’s *α* and McDonald’s *Ω*. Values of Cronbach’s *α* above 0.70 and McDonald’s Ω above 0.80 were considered acceptable (33). Criterion validity was evaluated by calculating Pearson’s correlation coefficients, classified as small (*r* ≈ 0.10), medium (*r* ≈ 0.30), and large (*r* ≈ 0.50) (34). Statistical significance was set at *p* < 0.01 (two-tailed). Discriminant validity of the scale was established using the heterotrait-monotrait (HTMT) ratio, with a cut-off point less than 0.85 (35).

## 3 Results

**Figure 2:**
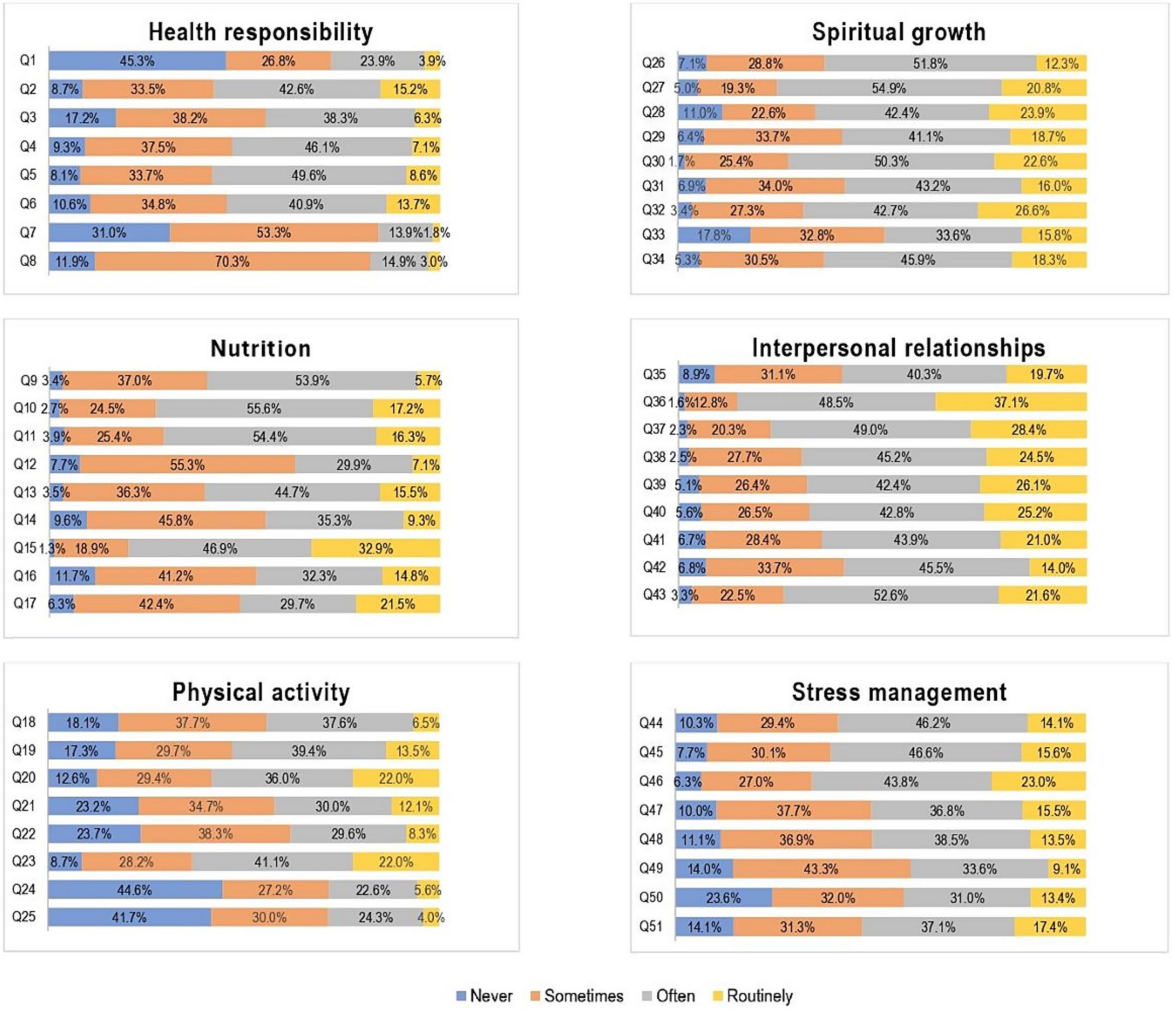
Response percentages of the participants (*N* = 827).

The following descriptive results are presented for sample characterization purposes and should be interpreted as preliminary. Figure 2 shows the percentage rates for each item among participants. No items demonstrated an absence for any given response. However, some responses indicated rates below 10.0%, including “routinely” as the least responded in HR and PA dimensions. The items in the N, SG, and SM dimensions revealed a prevalence of intermediate levels, characterized by responses of “sometimes” and “often.” Finally, items of the IR dimension were represented by responses to the highest-frequency levels, “often” and “routinely.”

### 3.1 Construct validity

**Figure 3:**
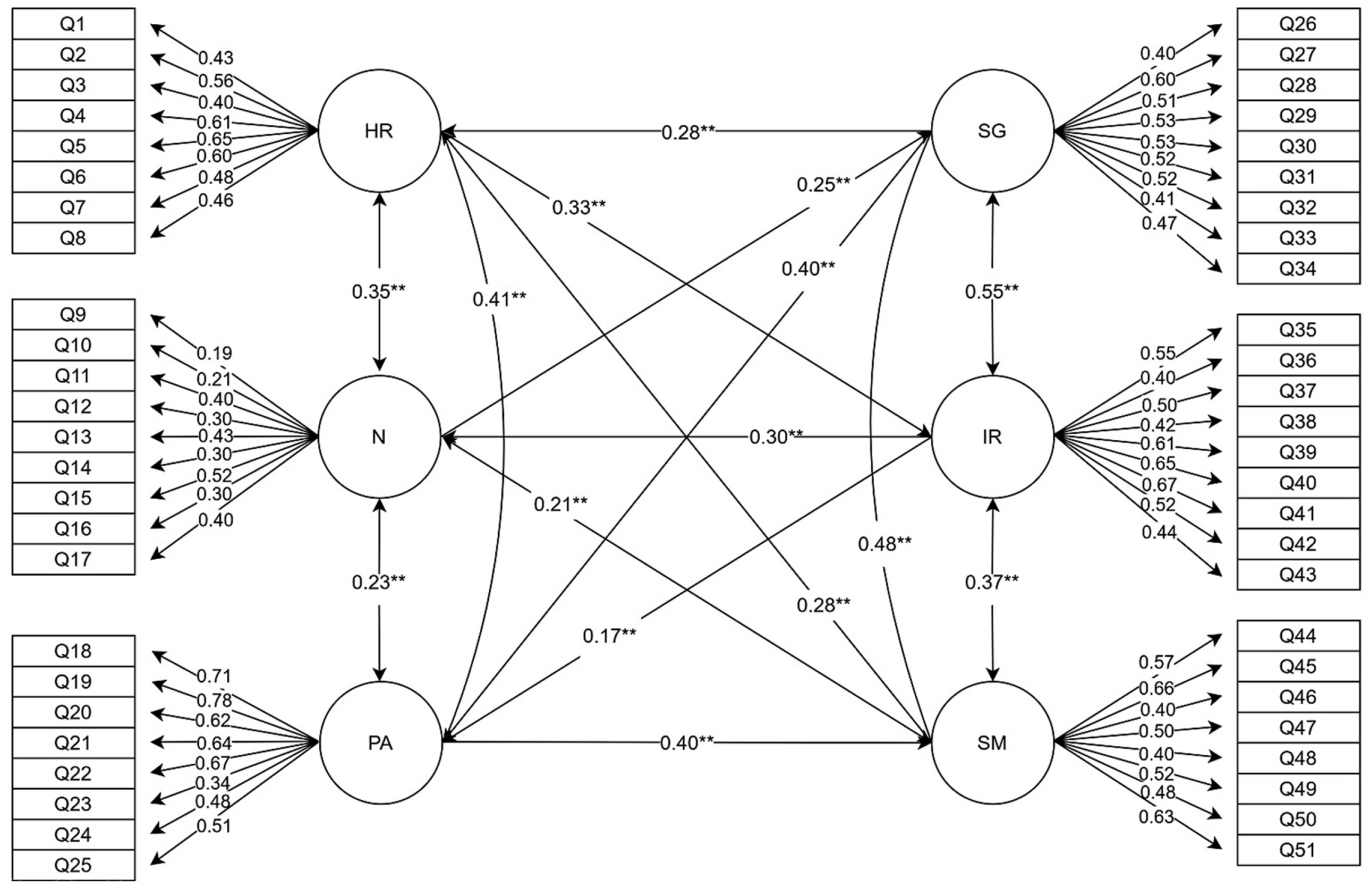
Structural diagram of the Mongolian version of the HPLP-II.

The initial CFA applied the six-factor measurement model. The Kaiser-Meyer-Olkin index (KMO = 0.90) and Bartlett’s sphericity test (*χ*^2^ = 14233.03, df = 1,275, *p* < 0.001) confirmed sampling adequacy for factor analysis. The goodness-of-fit indices were found to be acceptable (CFI = 0.95, GFI = 0.95, TLI = 0.95, RMSEA = 0.031, and SRM*R* = 0.065). Figure 3 presents the CFA results, including factor loadings and intercorrelation coefficients. Factor loadings ranged from 0.19 to 0.78. The factor loadings of the dimensions were as follows: 0.40–0.65 for HR, 0.40–0.60 for SG, 0.19–0.52 for N, 0.42–0.67 for IR, 0.40–0.66 for SM, and 0.34–0.78 for PA. Six items demonstrated lower factor loadings (five in N and one in PA).

All dimensions of the scale showed a significant, positive correlation (*p* < 0.01) with the overall HPLP-II scale, suggesting that health-promoting behaviors are interrelated. Correlations ranged from weak (PA with IR, *r* = 0.17) to moderate (SG with SM, *r* = 0.48). A strong positive correlation was observed between SG and IR (*r* = 0.55), reflecting a close relationship between personal development and social communication.

### 3.2 Internal consistency

Table 3 shows the internal consistency for the HPLP-II scale and its dimensions. Both McDonald’s omega (*ω* = 0.92) and Cronbach’s alpha (*α* = 0.91) demonstrated acceptable internal consistency for the overall scale. Most of the dimensions demonstrated acceptable reliability coefficients: HR (*ω* = 0.77; *α* = 0.77), PA (*ω* = 0.85; *α* = 0.84), SG (*ω* = 0.83; *α* = 0.84), IR (*ω* = 0.82; *α* = 0.82), and SM (*ω* = 0.79; *α* = 0.80). However, the N dimension showed questionable internal consistency, with the lowest ω and *α* coefficients of 0.42 and 0.56, respectively. This is an emerging finding that requires further examination.

**Table 3:** Internal consistency (McDonald’s *ω* and Cronbach’s *α*) and discriminant validity (HTMT ratio) of the Mongolian HPLP-II (51 items).

Dimensions	*ω*	*α*	HR	N	PA	SG	IR	SM
Health responsibility	0.77	0.77	-					
Nutrition	0.42	0.56	0.51	-				
Physical activity	0.85	0.84	0.48	0.41	-			
Spiritual growth	0.83	0.84	0.40	0.34	0.41	-		
Interpersonal relationships	0.82	0.82	0.40	0.40	0.20	0.65	-	
Stress management	0.79	0.80	0.27	0.29	0.46	0.56	0.42	-

### 3.3 Item-level analysis of the nutrition dimension

Given the low internal consistency of the nutrition (*α* = 0.56), we conducted item-level analyses to identify specific problematic items. Table 4 presents factor loadings, corrected item-total correlations, and Cronbach’s *α* if item deletes for each of the nine nutrition items.

**Table 4:** Item-level statistics for the nutrition dimension of the Mongolian HPLP-II.

Item	*λ*	Corrected Item-total correlation	*α* if item deleted
Q9	0.03	0.06	0.58
Q10	0.14	0.16	0.55
Q11	0.35	0.30	0.51
Q12	0.46	0.30	0.51
Q13	0.65	0.41	0.48
Q14	0.48	0.32	0.51
Q15	0.39	0.32	0.51
Q16	0.31	0.22	0.54
Q17	0.31	0.21	0.54

Factor loadings ranged from 0.03 to 0.65. The following items showed factor loadings below 0.35: Q9 (fat consumption, *λ* = 0.03), Q10 (sugar consumption, *λ* = 0.14), and Q13 (read food labels, *λ* = 0.31). Corrected item-total correlations were below 0.20 for Q9 (0.06) and Q10 (0.16). Removing Q9 and Q10 would increase Cronbach’s *α* from 0.56 to approximately 0.60.

### 3.4 Discriminant validity

The HTMT ratio correlations were used to examine the discriminant validity. All the HTMT values were below the recommended cut-off point of 0.85, which suggested the six factors achieved good discriminant validity (see Table 3).

## 4 Discussion

The purpose of the study was to translate and culturally adapt the HPLP-II and examine its construct validity and reliability among Mongolian undergraduate students. While the primary purpose of this study was to cross-culturally validate the HPLP-II in the Mongolian context, the response patterns observed in our sample offer preliminary, exploratory insights into health behaviors among participants. In this validation study, the highest frequency of “often” and “routinely” responses was observed in the IR and SG dimensions. Conversely, the dimensions HR and PA showed the highest proportion of “never and “sometimes” responses among participants, aligning with global concerns about sedentary lifestyles and poor self-health management among youth, are presented as preliminary descriptive findings from this validation sample (1). Future studies using the fully validated instrument are needed to confirm these patterns and to investigate their potential correlates. These interpretations are speculative as they are based on a convenience sample from a validation study. To confirm the conclusions drawn about the health behaviors of this population, further confirmatory research is required that uses a fully validated instrument and a more diverse sample.

The six-factor structure of the Mongolian HPLP-II was supported by the CFA, aligning with the original version of the HPLP-II (6). The fit indices of the overall scale met the established cut-off points for good fit (32), indicating that the theoretical model of health-promoting behaviors applies to the Mongolian context. Also, the findings align with previous validation studies across multiple cultural contexts, including Malay (14), Spanish (16), and Turkish (17), which reported comparable fit indices.

The internal consistency for the overall translated HPLP-II was excellent, aligning with the original version of the scale (*α* = 0.91) (6). All dimensions of the scale demonstrated acceptable reliability, except the nutrition dimension, which exhibited the lowest reliability, suggesting potential issues with internal consistency (*ω* = 0.42; *α* = 0.56).

Several factors may explain this low reliability. First, the nutrition items were substantially modified from the original HPLP-II to align with Mongolian national dietary guidelines (28), which changed the focus from general healthy eating habits to adherence to specific cultural standards. As Beaton et al. (27) state, ecological validity in cross-cultural adaptation may come at the cost of internal consistency. Second, the nutrition dimension consists of items measuring diverse behaviors, such as consumption of different food groups and reading food labels, which may reflect a broader, inherently less cohesive construct compared to other dimensions.

Consistent with this pattern, previous studies have reported Cronbach’s *α* to be lower than the accepted threshold of 0.70 in the nutrition subscale. Sousa et al. (8) found an *α* of 0.62 among Portuguese adolescents; Pérez-Fortis et al. (16) found Cronbach’s *α* = 0.68 among Spanish college students; Teng et al. (36) found an *α* of 0.69 among the Chinese population aged 19 to 70; Haddad et al. (37) found an *α* of 0.63 among Jordanian school students, teachers, and workers; and Carlson (38) found an *α* of 0.54 among US participants. These findings suggest that low reliability in the nutrition dimension is not unique to the Mongolian context but may represent a broader challenge in measuring diverse dietary habits with a limited set of items.

In addition, our initial factor analysis indicated that items Q9 (“fat consumption”) and Q10 (“sugar use”) should be removed. Thus, the Cronbach’s *α* coefficient would improve to 0.60 with these items removed. Furthermore, the discriminant validity of the Mongolian HPLP-II was acceptable, consistent with previous validation studies. For example, the Spanish (16) and Malay (14) versions of the HPLP-II scale reported that the HTMT ratio supported the cross-cultural validity of the six-factor structure.

Despite this low reliability, we retained all nine nutrition items in our study for the following reasons: (1) this is the first validation study of the HPLP-II in the Mongolian context, and the psychometric properties of the nutrition dimension cannot be assumed; (2) removing items based on a single sample would be premature; (3) retaining all items enables future studies to examine and further refine the instrument using independent samples; (4) despite its low internal consistency, the nutrition dimension remains conceptually important, as healthy eating is a core component of health-promoting lifestyles according to Pender’s Health Promotion Model (10). The adapted nutrition is functionally analogous to the original HPLP-II. However, low internal consistency suggests it may not be psychometrically equivalent. Additionally, experiential equivalence may be partially compromised.

Our study has several strengths, including a standard translation (27), cultural adaptation procedure following national guidelines (28), and a substantial sample size (*N* = 827).

Several limitations must be acknowledged. The employment of convenience sampling from three universities in Ulaanbaatar may not represent students across Mongolia, particularly in rural areas. It is common for students from provincial areas in Mongolia to migrate to Ulaanbaatar for university education, meaning our sample primarily represents students currently residing in the capital city. Furthermore, the findings may not generalize to students enrolled in private universities. Additionally, this study did not examine test–retest reliability or measurement invariance across key subgroups, such as gender, geographic origin, or year of study. Future studies should assess these aspects to establish the validity of the Mongolian HPLP-II scale. As previously indicated, the low reliability of the nutrition dimension suggests that the modified items require further revision or that the assessment of dietary habits may require a complementary, locally developed tool.

## 5 Conclusion

Our study provides the first validation evidence of the HPLP-II in samples within the Mongolian context. The findings support the six-factor structure, construct validity, and overall reliability of the scale for assessing health-promoting lifestyles among university students in Ulaanbaatar. While the overall scale demonstrated acceptable internal consistency, the nutrition dimension presented low reliability, highlighting a cultural and validation challenge that warrants further refinement. Particularly, items modified to align with the Mongolian national dietary guidelines may have compromised the internal consistency of the nutrition dimension, suggesting that future studies should either revise those items or develop a complementary, culturally appropriate nutrition module. Despite this limitation, the Mongolian HPLP-II remains a useful tool for designing and assessing evidence-based health promotion interventions in university settings, provided that the nutrition dimension is interpreted cautiously and prioritized for further validation in more diverse populations.

## Data Availability

The raw data supporting the conclusions of this article will be made available by the authors, without undue reservation.
