# Supplementary material for: Cross-cultural adaptation and validation of the health-promoting lifestyle profile II for Mongolian university students
Source: Front Public Health. 2026 Jun 5;14:1846655. doi: 10.3389/fpubh.2026.1846655 (PMC13279628; doi:10.3389/fpubh.2026.1846655)
Supplement: Supplementary file 1 [file Table_1.docx]

Supplementary Material

**Table 1.** Backward translation of the Mongolian HPLP-II (51 items)

| **Items** | | **N** | **S** | **R** | **O** |
| --- | --- | --- | --- | --- | --- |
| **Health responsibility** | | | | | |
| Q1 | I read or watch TV programs about improving health. |  |  |  |  |
| Q2 | I attend educational programs on personal health care |  |  |  |  |
| Q3 | I control my weight and inspect my body at least monthly for physical changes/danger signs |  |  |  |  |
| Q4 | I report any unusual signs or symptoms to a physician or other health professional. |  |  |  |  |
| Q5 | I discuss my health concerns only with health professionals |  |  |  |  |
| Q6 | I get a second opinion when I question my health care provider's advice. |  |  |  |  |
| Q7 | I confirm health professionals in order to understand their instructions |  |  |  |  |
| Q8 | I seek guidance or counseling when necessary |  |  |  |  |
| **Nutrition** | | | | | |
| Q9 | I consume 2 servings of fats each day (vegetable oil, butter, sour cream) |  |  |  |  |
| Q10 | I avoid consuming sugar |  |  |  |  |
| Q11 | I eat 9-10 servings of whole wheat bread, cereal, rice, and pasta each day |  |  |  |  |
| Q12 | I eat 2-4 servings of fruit each day |  |  |  |  |
| Q13 | I eat 3-5 servings of vegetables each day. |  |  |  |  |
| Q14 | I consume 3-4 servings of dairy product each day (milk, yoghurt, cheese, dried curd) |  |  |  |  |
| Q15 | I consume 3-4 servings of protein contained products each day (meat, fish, beans, nuts, eggs) |  |  |  |  |
| Q16 | I read labels to identify nutrients, fats, and sodium content in packaged food. |  |  |  |  |
| Q17 | I eat breakfast daily |  |  |  |  |
| **Physical activity** | | | | | |
| Q18 | I follow a planned exercise program |  |  |  |  |
| Q19 | I exercise vigorously for 20 or more minutes at least three times a week (such as brisk walking, bicycling, aerobic dancing, using a stair climber) |  |  |  |  |
| Q20 | I take part in light to moderate physical activity (such as sustained walking 30-40 minutes 5 or more times a week) |  |  |  |  |
| Q21 | I take part in leisure-time (recreational) physical activities (such as swimming, dancing, bicycling |  |  |  |  |
| Q22 | I do stretch exercises at least 3 times per week. |  |  |  |  |
| Q23 | I get exercise during usual daily activities (such as walking during lunch, using stairs instead of elevators, parking car away from destination and walking) |  |  |  |  |
| Q24 | I check my pulse rate when exercising |  |  |  |  |
| Q25 | I reach my target heart rate when exercising |  |  |  |  |
| **Spiritual growth** | | | | | |
| Q26 | I feel I am growing and changing in positive ways. |  |  |  |  |
| Q27 | I believe that my life has purpose |  |  |  |  |
| Q28 | I look forward to the future. |  |  |  |  |
| Q29 | I feel confident |  |  |  |  |
| Q30 | I work toward long-term goals in my life. |  |  |  |  |
| Q31 | I find each day interesting and challenging |  |  |  |  |
| Q32 | I am aware of what is important to me in life. |  |  |  |  |
| Q33 | I feel connected with some force greater than myself |  |  |  |  |
| Q34 | I expose myself to new experiences and challenges. |  |  |  |  |
| **Interpersonal relationships** | | | | | |
| Q35 | I discuss my problems and concerns with people close to me. |  |  |  |  |
| Q36 | I praise other people easily for their achievements. |  |  |  |  |
| Q37 | I maintain meaningful and fulfilling relationships with others |  |  |  |  |
| Q38 | Spend time with close friends |  |  |  |  |
| Q39 | I find it easy to show concern, love and warmth to others. |  |  |  |  |
| Q40 | I touch and am touched by people I care about |  |  |  |  |
| Q41 | I find ways to meet my needs for intimacy |  |  |  |  |
| Q42 | I get support from a network of caring people. |  |  |  |  |
| Q43 | I settle conflicts with others through discussion and compromise. |  |  |  |  |
| **Stress management** | | | | | |
| Q44 | I sleep and rest enough |  |  |  |  |
| Q45 | I take some time for relaxation each day. |  |  |  |  |
| Q46 | I accept those things in my life which I can not change. |  |  |  |  |
| Q47 | I concentrate on pleasant thoughts at bedtime I sleep well without thinking of unpleasant thoughts |  |  |  |  |
| Q48 | I use specific methods to control my stress |  |  |  |  |
| Q49 | I balance time between studying, work, and play |  |  |  |  |
| Q50 | I practice relaxation or meditation for 15-20 minutes daily. |  |  |  |  |
| Q51 | I balance my resting time to prevent tiredness |  |  |  |  |

*Note: N – never, S – sometimes, O – often, R – routinely*
